# Vesicoscopic vs. Open Ureteral Reimplantation According to Cohen and Leadbetter-Politano for Vesicoureteral Reflux

**DOI:** 10.3390/jcm12175686

**Published:** 2023-08-31

**Authors:** Christian Kruppa, Alexandra Wilke, Carola Hörz, Thomas Kosk, Tina Hörz, Guido Fitze, Katrin Schuchardt

**Affiliations:** Department of Pediatric Surgery, University Hospital Dresden, Technical University Dresden, 01307 Dresden, Germanyguido.fitze@uniklinikum-dresden.de (G.F.);

**Keywords:** vesicoscopy, pneumovesicoscopic operation, COHEN, LEADBETTER-POLITANO, ureteral reimplantation, vesico-ureteral-reflux

## Abstract

Background: The minimally invasive implementations of the established open methods for the correction of primary vesicoureteral reflux have proven to be successful in terms of feasibility and safety. The aim of this study was to investigate to what extent pediatric patients benefit from vesicoscopic operations. Methods: Between 2010 and 2022, 224 children (359 ureters) underwent ureteral reimplantation for vesicoureteral reflux in our clinic. Children, operated on according to the COHEN technique, underwent an open approach in 39 cases, whereas 151 patients were operated on vesicoscopically. A total of thirty-four children have received a ureteral reimplantation according to the LEADBETTER-POLITANO technique: twenty-nine openly and five vesicoscopically. The open and vesicoscopic groups were compared with regards to perioperative data and postoperative course. Results: The mean operating time was significantly shorter for open than for the vesicoscopic procedures in the COHEN group (99 vs. 149 min, *p* < 0.001). Similarly, a comparison of ureteral reimplantations, according to LEADBETTER-POLITANO, favored the open procedure, although this was not significant (161 vs. 196 min, *p* = 0.135). There was no significant difference in the recurrence rate of all the groups. All procedures remained within the accepted range with a success rate of at least 96%. In the postoperative course, a significantly shorter hospital stay (4.1 vs. 7.9 days, *p* < 0.001 for COHEN-patients; 5.6 vs. 9.2 days for LEADBETTER-POLITANO-patients), as well as a significantly lower need for continuous analgesic administration, was observed for the vesicoscopic approaches of both methods (0.8 days in both vesicoscopic groups vs. 3.7 resp. 3.8 days in open groups, *p* < 0.001). In addition, the time of bladder drainage was significantly shorter in open techniques (7.2 vs. 1.9 days, *p* < 0.001 for COHEN-patients; 3 vs. 8.7 days for LEADBETTER-POLITANO-patients). Conclusions: For almost all underlying causes, the surgical treatment of vesicoureteral reflux can be performed vesicoscopically, even if bilateral, in one session. Patients benefit significantly from the use of minimally invasive surgery in the postoperative course with faster mobilization, less need for analgesics, a shorter bladder drainage and a reduced hospital stay, compared with its open counterparts.

## 1. Introduction

With a prevalence of 0.4–1.8%, vesicoureteral reflux is one of the most common congenital malformations. In up to 20% of prenatally diagnosed hydronephroses and in almost half of the children with febrile urinary tract infections, vesicoureteral reflux is detected [1,2]. Therapy covers a wide spectrum from conservative waiting, with or without antibiotic prophylaxis, to cystoscopic injection and surgical therapy. The complexity of decision-making leads to increasingly divergent expert opinions, depending on the age of children, the grade of vesicoureteral reflux and especially on the subjective preferences of parents and therapists [3,4].

Untreated reflux can lead to recurrent pyelonephritis and renal damage. While low-grade reflux in young children often matures or is amenable to injection therapy, surgical correction is the treatment of choice for persistent dilating reflux, recurrent urinary tract infections or renal parenchymal scarring. Vesicoureteral reflux is one of the most frequent indications for urological surgery in children [5,6].

Open procedures require a sufficiently large skin incision to reach the surgical site. In addition, the open transvesical procedures require an opening of the bladder. This leads to increased postoperative wound pain and, additionally in the case of transvesical procedures, to slight postoperative bleeding into the bladder, possible bladder spasms and micturition difficulties. Thus, minimally invasive adaptations of the most popular open methods have already been successfully introduced to avoid the disadvantages of access routes.

Yeoung et al. first presented a series of patients who underwent COHEN pneumovesicoscopic surgery in 2005. Since then, the feasibility of this procedure has been validated in a number of studies [7,8,9]. In the presence of megaureters, the COHEN procedure is not suitable due to insufficient submucosal tunnel length. The same applies if the ureter cannot be mobilized sufficiently. In this case, the minimally invasive version of ureteral reimplantation according to LEADBETTER-POLITANO is suitable. Both methods can be performed safely on both sides in one session [10,11,12,13].

Due to the pneumovesicoscopic approach and the small surgical area, the above-mentioned minimally invasive procedures are considered technically more demanding than the minimally invasive implementation of the extravesical antirefluxive surgery according to LICH-GREGOIR [14]. The laparoscopic or robotic feasibility of LICH-GREGOIR antirefluxplasty is well documented [15,16]. Compared with open LICH-GREGOIR surgery and other vesicoscopic techniques, the minimally invasive nature of its transperitoneal approach is less evident. In addition, the cutting of the bladder musculature performed in the LICH-GREGOIR method can result in a transient postoperative bladder emptying disorder, especially in bilateral surgery, which reduces acceptance [17,18].

## 2. Materials and Methods

This retrospective study included 224 children, 150 of them girls, who underwent ureteral reimplantation according to COHEN (*n* = 190) or LEADBETTER-POLITANO (n = 34) for vesicoureteral reflux in our hospital between 2010 and 2022. All children were characterized for sonography, urine status, urine culture, renal function from MAG3-scintigraphy and postoperative course. Patients were diagnosed by voiding cystourethrogram or contrast-enhanced voiding urosonography. In total, 25 (11%) of the 224 children had already undergone previous operations, such as endoscopic injection, ureterocele slitting or insufficient antirefluxive surgery. Patients were excluded if they simultaneously underwent other extensive therapies, e.g., bladder augmentation.

The average age of the children was 3.9 years (1 month–17.6 years). In 135 patients, both sides were operated on, and a total of 359 ureteral units (including 28 double ureters) were implanted (Table 1 and Table 2). 

The indication for ureteral reimplantation was high-grade dilating vesicoureteral reflux with recurrent urinary tract infections or its persistence with functional impairment according to the AUA guidelines [19]. Table 3 and Table 4 show the underlying diagnoses. Written parental consent was obtained for all operations. The local ethic committee provided ethical approval.

Based on anatomical conditions, the choice of surgical method was made. The COHEN technique was preferred unless a ureter was too short or the achievable tunnel length did not match the ureteral diameter. In this case, LEADBETTER-POLITANO surgery was performed. The choice between open and vesicoscopic surgery was based on the preferences of surgeons and the level of experience in the implementation of vesicoscopy. 

The occurrence of bilateral vesicoureteral reflux with unilateral megaureter was found in two vesicoscopically treated patients. In both children, the megaureter was reimplanted according to LEADBETTER-POLITANO and the ureter of the opposite side according to COHEN.

- Surgical techniques
  ⚬ The open access to the bladder

The patient is operated on in a supine position. The anterior bladder wall is accessed via a suprapubic Pfannstiel incision with transverse skin incision and longitudinal opening of the fascia extraperitoneally. Then, the anterior bladder wall is longitudinally opened.

  ⚬ Creating the pneumovesicum for vesicoscopic surgery

The patient is placed in a supine position with legs slightly bent and spread. Under cystoscopic control, a 5-mm trocar is suprapubically inserted in the center and two 3.9-mm trocars are placed in the right and left of the first one via small incisions in a skin fold of the lower abdomen. To avoid trocar deplacement, the bladder is pexed to the abdominal wall with penetrating sutures in the area of the incisions beforehand. In vesicoscopic LEADBETTER-POLITANO surgery, it is important that the trocars are placed further cranial than the new bladder wall passage of the ureter. This should be assessed preoperatively, for example, in patients with a megaureter or after previous surgery. 

Afterwards, the cystoscope is removed and the 5-mm optic is inserted. The irrigation solution is replaced with CO_2_ at 7 mmHg. For stable camera guidance, it is important to attach the optic to a holding device. During vesicoscopic surgery, the patient should be under muscle relaxants continuously.

- Ureteral reimplantation
  ⚬ The COHEN-procedure

In both access routes, subsequent ureteral reimplantation largely follows the principle described by COHEN [20]. The affected ureters are intubated and electrically exposed. They must be mobilized until they can be placed tension-free to the opposite side.

In cases of bilateral ureteral reimplantation, a submucosal tunnel, approximately 3 to 5 cm long, is prepared between the original ureteral entry points, depending on the anatomy and age of the child. Both ureters are passed through this tunnel to the respective opposite side. After ispilateral pexia of the ureters at the bladder wall, the neoureterostia are then sutured contralaterally with 5/0 vicryl with prior minor shortening. In unilateral surgery, the site of neoostium is defined by a small mucosal incision contralaterally, about 1 cm cranial to the opposite ostium and tunneled there. Splinting of the ureters was performed only in children with renal insufficiency or in patients with bilateral vesicoureteral reflux and markedly hydronephrotic kidneys.

  ⚬ The LEADBETTER-POLITANO-procedure

For both access routes, the operation begins, as described above, with the mobilization of the ureter to be operated [21]. The ureter is electrically dissected under traction intravesically, for at least the desired tunnel length. The ureter forms on the bladder wall under traction on the retaining suture. In this course, the bladder wall is incised according to the required tunnel length at least 3–4 cm cranial to the original ostium. From here, the submucosal tunnel is subtly dissected towards the original ureterostium. 

Subsequently, during the vesicoscopic approach, dissection is performed through the new bladder incision in the direction of the original ureteral entrance behind the bladder wall. The ureter is found here and guided cranially through the bladder wall. 

In open surgery, the ureter is also presented extravesically. In both methods, the ureter is then pulled through the prepared submucosal tunnel and fixed to the bladder muscle at the wall passage. Finally, the neoostium is sutured into orthotopic place after the ureter has been slightly shortened. If necessary, the opposite side is now operated in the same way or according to COHEN during the same session. Reimplanted ureters are splinted transcutaneously with 4 Fr probes, which may be removed after 6 days on an outpatient basis.

- The completion of operation

Finishing a vesicoscopic reimplantation, the bladder is drained by a foley catheter for 1 to 2 days, irrespective of whether ureteral splinting was performed. After removal, trocar entrances are closed only with plaster strips.

In case of open reimplantation of the ureter, the bladder is closed by a continuous suture after insertion of a cystofix catheter, and further wound closure is carried out in layers in the typical manner. The suprapubic bladder drainage can be removed after a week.

Postoperative pain therapy was performed with metamizole, ibuprofen and paracetamol, according to our general standard, and was based exclusively on the patient’s clinical condition. Postoperative reflux diagnostics with voiding cystourethrogram or contrast-enhanced voiding urosonography was performed only in patients with urinary tract infections or unclear sonographic findings.

All children remain under regular control by us and local pediatric nephrologists until adolescence. Postoperatively, prophylactic antibiotics are administered for 4 weeks.

## 3. Results

In all patients in whom the creation of the pneumovesicum was successful, the vesicoscopic operation could also be performed completely. In two patients, conversion to the open procedure was necessary at the early beginning of the learning curve of the vesicoscopic COHEN operation because the trocars could not be placed.

### 3.1. COHEN-Patients

The operation time of the vesicoscopic surgery according to COHEN proves to be significantly longer than the operation time of open surgery and the learning curve shows only a modest downward slope. For the first 40 operations, with an average duration of 179 min, there is only a minor correlation with an increasing number of procedures with a Spearman’s correlation factor of rsp = −0.31 (α = 0.05). The last 100 operations lasted about 137 min, and the last 40 were 124 min. The fastest vesicoscopic COHEN operation, 67 min, almost met the time of the shortest open one, 61 min. On average, we required 96 min for open unilateral and 100 min for bilateral COHEN surgery. Vesicoscopically, 126 min were required for unilateral and 163 min for bilateral approach.

In a comparison of the children operated on openly according to COHEN, in the vesicoscopic group, the time of bladder decompression via cystofix or foley catheter was significantly longer with an average of almost four times the drainage time in the open group. In a similar ratio, the need for continuous analgesic medication was significantly shortened (Table 5). This was followed by discontinuous oral pain medication on demand, on average, on the first operative day in the vesicoscopic group.

In addition, the time to discharge home for children who underwent vesicoscopic surgery was almost halved.

There were no significant differences in complications between the two groups. Among those operated on vesicoscopically, there were four children with micturition problems after foley catheter removal. The cause was a prevesical extravasation, which was treatable with a new bladder drainage for 5 days. This problem did not occur after increasing the minimum duration of bladder relief to 2 days. 

One patient, each with postoperative hydronephrosis, from the open and vesicoscopic groups, was treated by temporary percutaneous nephrostomies. Of the two other children operated vesicoscopically, one could be treated conservatively, while the other required surgical ureteral splinting due to a postoperative urinary tract infection.

In five vesicoscopically treated patients (six ureters), vesicoureteral reflux recurrences were diagnosed after recurrent urinary tract infections. This corresponds to 2.5% of the reimplanted 241 ureters. The ureters were found dislocated to their initial position. After implementing an ipsilateral ureteropexy on the bladder muscle, no recurrence has occurred so far (Table 5).

### 3.2. LEADBETTER-POLITANO-Patients

The comparison of the groups of children operated on according to LEADBETTER-POLITANO shows a greater average time for vesicoscopic procedures, although this was not statistically significant. Unilateral open surgery lasted an average of 142 min and bilateral 182 min. This compares with 188 and 227 min for vesicoscopic unilateral and bilateral procedures, respectively.

The duration of catheters relieving the bladder was significantly shorter in favor of the children who underwent vesicoscopic surgery. The postoperative time during which pain medication had to be administered at least three times daily was also significantly less for this group. This correlates with a 5.6 day shorter time to hospital discharge for the patients operated on in a minimally invasive way (Table 6).

In a young boy with primary vesicoureteral reflux into a megaureter who underwent open surgery at the age of 1.3 years, a recurred reflux 1° was found in the control voiding cystourethrogram, which remained uncomplicated and under conservative treatment without consequences.

## 4. Discussion

In recent years, with the ongoing development of minimally invasive procedures, the spectrum of surgical treatment options for vesicoureteral reflux has changed substantially. The required surgical access is achieved via smaller abdominal wall, and possibly bladder incisions, which leads to the expectation of being able to improve blood loss, postoperative pain and cosmetics. 

Pneumovesicoscopic procedures reduce the disadvantages of postoperative bleeding, the need for prolonged bladder drainage with associated spasms and irritation and the pain that can occur during micturition after catheter removal compared with open variants [22]. The assumption is that by implementing the minimally invasive LEADBETTER-POLITANO surgery, any pathology leading to vesicoureteral reflux can be successfully operated vesicoscopically, in addition to the already established COHEN technique. Thus, vesicoscopic procedures can also be performed in case of problematic tunnel preparation, such as after ureteroceles slitting or in megaureters. 

The advantage of the minimally invasive laparoscopic approach of extravesical antirefluxplasty according to LICH-GREGOIR is based on the avoidance of a larger abdominal incision. However, this then changes from an extra- to a transperitoneal approach. The principle-immanent necessity of detrusor incision remains unchanged. Thus, the risk of a transient bladder emptying disorder remains with the recommendation not to operate on both sides in one session. In addition, the operation of large megaureters is evaluated with restraint [14].

An increasing number of publications on COHEN pneumovesicoscopic ureteral reimplantation can be taken as a clear sign that this complex procedure has passed the test of time in terms of feasibility and safety [8,10,12,13,23]. Our success rate in children operated vesicoscopically after COHEN was 96% of patients (97.5% of ureters), which meets the range described for open procedures [24].

The regular splinting of reimplanted ureters is not necessary with the COHEN method. However, if there is evidence of marked renal dysfunction, this should be performed for at least 6 days to avoid postoperative hydronephrosis and possible postrenal failure in these children.

The operating time was significantly shorter in the open COHEN group with an average of 99 min than in the vesicoscopically operated group with 149 min (124 min for the last 40 patients). In this analysis, unilateral and bilateral interventions are combined with almost equal proportions in both groups. The minimum times are almost the same with 61 vs. 67 min (open vs. vesicoscopic). In comparison with the literature, our operating time for vesicoscopic ureteral reimplantation according to COHEN, with 126 min for one side and 163 min for both sides, is significantly below the data from a recent meta-analysis with 155 and 194 min, respectively [14]. 

This disadvantage of vesicoscopy, which is tolerable in everyday life, is all the more insignificant when the time of continuous analgesic administration is evaluated. In this regard, the group of children operated on according to COHEN vesicoscopically required only 0.8 days of regular pain medication on average, compared with 3.7 days for those operated on openly (*p* < 0.001). Less pain due to the minimally invasive approach, as well as the removal of the bladder catheters after 1.9 days vs. 7.9 days (vesicoscopic vs. open, *p* < 0.001), led to faster mobilization and subjective well-being in these children. This correlates with the significantly lower hospitalization time in the first group of 4.1 vs. 7.9 days (*p* < 0.001). 

For patients for whom the COHEN method seems inadequate, the LEADBETTER-POLITANO ureteral reimplantation technique is an option. Recently, there has been a number of publications on its successful pneumovesicoscopic application [10,11]. As described in these publications, it has also been our experience that the transvesical finding of the ureter takes most of the additional time. Nevertheless, the difference remained moderate with 196 min for the vesicoscopic operations compared with 161 min for the open ones. The small number of cases in our series is certainly a limitation. 

In principle, our group of patients operated on according to LEADBETTER-POLITANO only includes children in whom a decision was made against COHEN reimplantation due to more difficult anatomical conditions. In such a group, longer operation times are to be expected with more complex preparation, even after the learning curve has flattened out.

With the same access routes, the postoperative course of the LEADBETTER-POLITANO-patients was similar to those of the COHEN-patients. Despite percutaneous ureteral catheters, the children who underwent this type of vesicoscopic surgery required a continuous analgesic administration for only 0.8 days compared with 3.8 days for the open LEADBETTER-POLITANO group (*p* = 0.003). The faster mobilization of patients undergoing vesicoscopic surgery is reflected in the significantly shorter hospital stay of 5.6 vs. 9.2 days.

These results confirm and emphasize that patients benefit directly from the use of minimally invasive vesicoscopic procedures. The possibility to perform ureteral reimplantation according to COHEN or LEADBETTER-POLITANO, as required, completes the vesicoscopic approach. 

Both methods differ with regard to their indications. As is statistically supported in Table 4, the COHEN procedure was used in our study for simpler ureteral pathology, while operations according to LEADBETTER-POLITANO were only performed if the previously mentioned principle was not technically promising. Thus, the two methods are available side by side and not as alternatives and allow vesicoscopic management in one session of almost all reflux patients requiring surgery.

In two children with bilateral reflux and unilateral megaureter, we were able to combine both methods without any problems. Principally, any situation with vesicoureteral reflux patients should be treatable in a vesicoscopic way. 

For ureteral reimplantation, we believe that vesicoscopy satisfies the principle of minimal invasiveness to the greatest extent possible. Nevertheless, surgery via the vesicoscopic approach will have a reasonably difficult time spreading outside of specialized centers, since the technique is only assigned to limited indications and alternatives exist with open or laparoscopic procedures. Interesting further areas of application emerge, for example, the fenestration of ectopic ureteroceles or the resection of bladder diverticula [25].

Until now, the use of robotic systems for vesicoscopic applications has been limited by the size of the trocars. The first successful applications have already been reported [26]. The development of smaller instruments or single port systems will open up new perspectives in the future.

## 5. Conclusions

All the requirements of the surgical therapy of vesicoureteral reflux with negligible blood loss, minimal pain, rapid mobilization, shorter bladder drainage and reduced hospital stay can be met by using COHEN and LEADBETTER-POLITANO vesicoscopic ureteral reimplantation without sacrificing success rates or patient safety. 

The applicability of both methods extends the spectrum of vesicoscopic reflux surgery to almost all underlying anatomical situations. The clear structuring of the vesicoscopic procedures provides an excellent tool for junior training.

## Figures and Tables

**Table 1:** COHEN-patients.

	Open	Vesicoscopic	*p*-Value
Patients	39	151	
Mean age (years, SD)	2.4 (2.2)	4.3 (3.3)	< 0.001 *
Female/Male	20/19	113/38	0.017 **
Ureters per operation	1.8	1.7	0.071 *
Operation unilateral	9	61	0.136 **
Operation bilateral	30	90	0.136 **
Double ureters reimplanted	3	16	0.865 **
Mean grade of reflux ***	4.2	3.9	0.088 *

* Mann–Whitney U-test, ** Chi^2^-test, *** Counting the highest implanted grade.

**Table 2:** LEADBETTER-POLITANO-patients.

	Open	Vesicoscopic	*p*-Value
Patients	29	5	
Mean age (years, SD)	3.6 (3.3)	3.8 (2.2)	0.420 *
Female/Male	15/14	2/3	0.889 **
Ureters per operation	1.7	1.6 ***	0.671 *
Operation unilateral	17	2	0.741 **
Operation bilateral	12	3 ***	0.741 **
Double ureters reimplanted	9	0	0.348 **
Mean grade of reflux ****	3.5	3.8	0.085 *

* Mann–Whitney U-test, ** Chi^2^-test, *** two of them Cohen on opposite side, **** Counting the highest implanted grade.

**Table 3:** Methods and operational accesses by underlying pathology for the individual ureters.

	COHEN		Leadbetter-Politano	
	Open	Vesicoscopic	Open	Vesicoscopic
Ureteral units	69	243	41	6
Single ureter	45	216	12	5
Ureter duplex *	3	17	8	-
Ureter fissus	4	7	2	-
Secondary reflux **	5	1	4	-
Post injection	8	2	7	-
Redo operation	3	-	4	-
Ureterocele after slitting	1	-	4	1

* one ureter duplex means one ureteral unit, ** single ureter in neurogenic bladder.

**Table 4:** Methods according to underlying pathology for the individual ureters.

	COHEN	Leadbetter-Politano	*p*-Value
	Total	Total	Chi^2^-Test
Ureteral units	312	47	-
Single ureter	259	19	<0.001
Ureter duplex *	20	8	0.041
Ureter fissus	11	2	0.969
Secondary reflux	6	4	0.038
Post injection	10	7	0.002
Redo operation	3	4	0.002
Ureterocele after slitting	1	5	<0.001

* one ureter duplex means one ureteral unit

**Table 5:** Results of the COHEN patients.

Title 1	Open	Vesicoscopic	*p*-Value
Patients	39	151	
Operation time (minutes) SD	99 (36)	149 (47)	<0.001 *
Time of bladder drainage (d)	7.2	1.9	<0.001 *
Ureter splinting (n patients, %)	9 (23%)	5 (3%)	<0.001 **
Painkiller 3×daily (d)	3.7	0.8	<0.001 *
Hospital stay (d)	7.9	4.1	<0.001 *
Follow-up period (years)	8.1	4.6	<0.001 *
Recurrence (n patients, %)	0	6 (4%)	0.449 **
Paravasation (n patients)	1	4	1 **
Hydronephrosis (n patients)	1	3	0.975 **

* Mann–Whitney U-test, ** Chi^2^-test.

**Table 6:** Results of the LEADBETTER-POLITANO-patients.

Title 1	Open	Vesicoscopic	*p*-Value
Patients	29	5	
Operation time (minutes) SD	161 (46)	196 (50)	0.135 *
Time of bladder drainage (d)	8.7	3	<0.001 *
Painkiller 3×daily (d)	3.8	0.8	0.003 *
Hospital stay (d)	9.2	5.6	<0.001 *
Follow-up period (years)	7.2	1.4	<0.001 *
Recurrence (n, %)	1 (4%)	0	0.668 **
Paravasation (n patients)	0	0	-
Hydronephrosis (n patients)	0	0	-

* Mann–Whitney U-test, ** Chi^2^-test.

## Data Availability

Not applicable.
